# Extreme Drought Affects Visitation and Seed Set in a Plant Species in the Central Chilean Andes Heavily Dependent on Hummingbird Pollination

**DOI:** 10.3390/plants9111553

**Published:** 2020-11-12

**Authors:** Mary T. K. Arroyo, Valeria Robles, Ítalo Tamburrino, Jaime Martínez-Harms, René D. Garreaud, Paola Jara-Arancio, Patricio Pliscoff, Ana Copier, Jonás Arenas, Joaquín Keymer, Kiara Castro

**Affiliations:** 1 Facultad de Ciencias, Universidad de Chile, Las Palmeras 3425, 7800003 Ñuñoa, Santiago, Chile; vale.robles.j@gmail.com (V.R.); italobioambiental@gmail.com (Í.T.); ana.copier@ug.uchile.cl (A.C.); jonas.arenas@ug.uchile.cl (J.A.); joaquin.keymer@ug.uchile.cl (J.K.); kiara.castro@ug.uchile.cl (K.C.); 2 Instituto de Ecología y Biodiversidad (IEB), Las Palmeras 3425, 7800003 Ñuñoa, Santiago, Chile; paolajara@uchile.cl; 3 INIA, La Cruz, Instituto de Investigaciones Agropecuarias, Chorrillos 86, 2280454 La Cruz, Chile; jaime.martinez@inia.cl; 4 Departamento de Geofísica, Universidad de Chile, Avenida Blanco Encalada 2002, 8370449 Santiago, Chile; rgarreau@uchile.cl; 5 Centro de Ciencia del Clima y la Resiliencia (CR2), Avenida Blanco Encalada 2002, Universidad de Chile, 8370449 Santiago, Chile; 6 Departamento de Ciencias Biológicas y Departamento de Ecología y Biodiversidad, Facultad de Ciencias de la Vida, Universidad Andrés Bello, Republica 252, 8370134 Santiago, Chile; 7 Departamento de Ecología, Facultad de Ciencias Biológicas, Pontificia Universidad Católica de Chile, Alameda 340, 8331150 Santiago, Chile; pliscoff@uc.cl; 8 Instituto de Geografía, Facultad de Historia, Geografía y Ciencia Política, Pontificia Universidad Católica de Chile, Avenida Vicuña Mackenna 4860, 7820436 Macul, Santiago, Chile

**Keywords:** central Chile, extreme drought, floral longevity, floral resources, hummingbird-pollination, *Mutisia subulata*, *Oreotrochilus leucopleurus*, visitation rates, seed set

## Abstract

Rising temperatures and increasing drought in Mediterranean-type climate areas are expected to affect plant–pollinator interactions, especially in plant species with specialised pollination. Central Chile experienced a mega drought between 2010 and 2020 which reached an extreme in the austral summer of 2019–2020. Based on intensive pollinator sampling and floral studies we show that the subalpine form of *Mutisia subulata* (Asteraceae) is a specialised hummingbird-pollinated species. In a two-year study which included the severest drought year, we quantified visitation frequency, flower-head density, flower-head visitation rates, two measures of floral longevity, nectar characteristics and seed set and monitored climatic variables to detect direct and indirect climate-related effects on pollinator visitation. Flower-head density, nectar standing crop and seed set were significantly reduced in the severest drought year while nectar concentration increased. The best model to explain visitation frequency included flower-head density, relative humidity, temperature, and nectar standing crop with highly significant effects of the first three variables. Results for flower-head density suggest hummingbirds were able to associate visual signals with reduced resource availability and/or were less abundant. The negative effect of lower relative humidity suggests the birds were able to perceive differences in nectar concentration. Reduced seed set per flower-head together with the availability of far fewer ovules in the 2019–2020 austral summer would have resulted in a major reduction in seed set. Longer and more intense droughts in this century could threaten local population persistence in *M. subulata.*

## 1. Introduction

Over the past decades, all five Mediterranean-type climate areas of the globe have experienced severe droughts [1,2,3,4,5,6,7,8,9,10]. While dendrochronological studies show that droughts have long been a recurrent feature of Mediterranean-type climate ecosystems [3,11], the most recent episodes appear to have been exacerbated by increasing global temperatures and model-based projections of climate indicate that drying and warming will continue during the 21st century. Thus, current droughts in Mediterranean-type climate areas integrate two distinct climatic components.

Reflecting the general trends in Mediterranean-type climate areas, central Chile has seen a consistent decline in annual precipitation since the late 1980s, at least partially attributable to anthropogenic forcing [12]. This trend was exacerbated during the last decade when annual rainfall fell below the long-term mean across the region every year. Up until 2018 the rainfall deficit during the so-called central Chile “mega drought” ranged from 25 to 50% (relative to the 30-year mean for the years 1980–2010) with detrimental impacts on surface hydrology [2]. The winter of 2019 further stood out as one of the four driest in the centennial record [13] and record high temperatures were experienced over the following spring and summer. From 2016 to 2018 onwards, browning of the foliage of sclerophyllous forest trees became widespread [14] and high elevation cushion plants in the Andes dried out much earlier than usual.

The combination of marked precipitation deficits and rising global temperatures in Mediterranean-type climate areas such as central Chile is predicted to affect plant–pollinator interactions and plant fitness, given that pollinator activity is well known to be affected both directly and indirectly by temperature and water stress [15,16]. Outcrossing species that require external pollinators are expected to be affected more severely than species capable of autonomous self-pollination, and species with specialised pollination, more than species with generalised pollination. Although many studies have addressed the effects of drought and temperature on specific aspects of plant–pollinator interactions, studies on how water and temperature stress integrally impact plant–pollinator interactions and fitness are still scarce [16,17,18,19,20]. Pollinator activity is well known to be directly affected both positively and negatively by changes in ambient temperature [21,22,23,24,25,26,27,28,29,30,31,32]. On the other hand, drought is known to cause mortality in hummingbirds, for example [33]. Nevertheless, responses to increased temperature and precipitation deficits are likely to vary among functional groups in accordance with their specific temperature optima and different body sizes (e.g., insects versus hummingbirds), as well as in relation to ecological context making broad generalizations difficult [34,35].

With regard to indirect drivers, pollinators are strongly sensitive to floral density [36,37] which they use as a cue to the availability of floral resources. Floral density ultimately [27] depends on the amount of germination in annual species and renewed or continued vegetative growth in perennial species, which are largely a function of the amount of soil moisture and ambient temperature, importantly integrated over previous years when it comes to perennial species. Under drought conditions, independently of increased temperature, floral resources are likely to be reduced [38], affecting visitation frequency (but not necessarily flower-visitation rates) [34]. Water stress is known to reduce nectar volume [39,40], while warmer and drier weather can reduce nectar standing crop on account of higher levels of evaporation [41] and lead to secretion in fewer flowers [42,43]. More viscid nectar could potentially affect extraction ability in some pollinator groups. Other expected indirect effects of drought in combination with warmer global temperatures relevant to pollinator activity include reduced corolla sizes [16,19,20] and reduced potential flower longevity as a result of more rapid flower development under warmer temperatures [44,45] and accelerated floral senescence as a result of soil drying and water stress [45].

Here, we take advantage of the extreme drought conditions in 2019 (preceded by an unusually long period of precipitation deficits) and the ensuing warm summer of 2020, to investigate the impacts of drought on pollinator activity and plant fitness in the subalpine form of *Mutisia subulata* (Asteraceae). Characterised by large red flower-heads typically associated with bird-pollination, hummingbirds have been casually seen on *M. subulata* [46]. Visits by insects and more specifically by bees and butterflies have also been mentioned [46,47]. *M. subulata* thus provides an interesting potential model for determining the effect of extreme drought on bird pollination in the Mediterranean-type climate area of central Chile.

In this paper, we first (1) analyse climate and snow line data over the past decades in order to contextualize our study. We then (2) present direct and indirect evidence for a high level of dependence of the subalpine form of *M. subulata* on hummingbird-pollination. Having shown without doubt that *M. subulata* is strongly dependent on hummingbirds for pollination, we go on to (3) ask whether hummingbird visitation and seed set in *M. subulata* were reduced in the extremely dry and warmer 2019–2020 austral summer compared to the previous year. Finally, with the objective of detecting the drivers of predicted changes in pollinator visitation under severe drought in the focal species, we (4) modelled the effect of temperature, wind speed, cloud cover, floral abundance, and nectar availability on visitation frequency based on data obtained over the same period of the summer season in the two years.

We show *M. subulata* to be a strongly pollinator-dependent, specialised hummingbird-pollinated species. We found that although warmer temperatures favoured hummingbird visitation in the cool subalpine setting, the negative effects of greatly reduced floral abundance and lower relative humidity (the last, seemingly affecting nectar concentration) in the driest year were far greater than the positive effects of warmer temperatures, leading to a pollination deficit and reduced seed set per flower-head. The overall reduction in seed set in the driest year, taking into account the many fewer available ovules due to reduced floral abundance, was huge.

## 2. Results

### 2.1. Characterization of the Central Chilean Mega-Drought and the Extreme Drought of 2019

**Figure 1:**
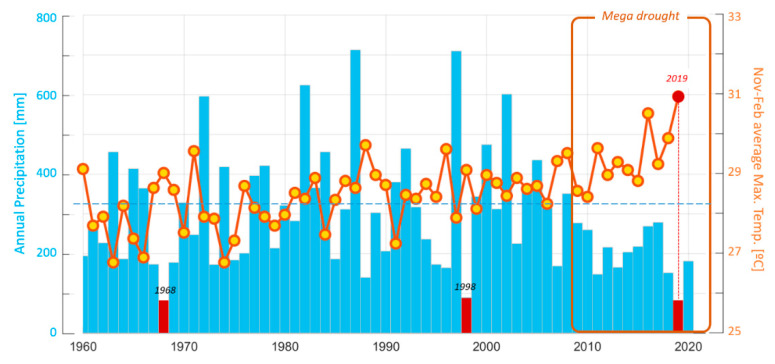
Variability and trends in central Chile’s hydroclimate since 1960. The light-blue bars are the annual precipitation for the city of Santiago (Quinta Normal) from 1960 up to the early spring of 2020 (and hence including the previous winter months when most precipitation is received). The horizontal dashed line is the long-term mean. Extreme drought years for the period (highly reduced precipitation in the winter months) of 1968, 1998 and 2019 are highlighted in red. The central Chile mega drought (from 2010 onwards) is also identified. The orange line with yellow circles gives the average daily maximum temperature over the late spring–early summer months (November–December) for a given calendar year and the mid–late summer months (January–February) for the following calendar year (so as to capture the austral summer). All values are plotted with respect to the calendar year of November and December The last summer season (November–December 2019, January–February 2020) is highlighted by the red dot, which according to the convention adopted, is plotted for the year 2019. The last value is representative of the 2020 austral summer. Data source: downloaded from the CR2 climate explorer (http://explorador.cr2.cl/).

Figure 1 depicts year-to-year variability in annual precipitation for Santiago, Chile, representative of the latitude of our study site. Superimposed on this variability, rainfall exhibited a weak decline as of the late 1980s. As of 2010, a long dry period set in, referred to as the central Chile mega drought [2]. Considering the entire period of the mega drought, the year 2019 stands out, with merely 85 mm of rainfall accumulated in Santiago, a quarter of the long term average for the period (1980–2010). This condition affected all of central Chile (30–35° S) with rainfall deficits between 70–90% with respect to 1980–2010 and a 12 month standardised precipitation index (SPI) below −2.5 [13] which is considered extreme. The average daily maximum temperatures for the spring and summer months between November 2019 and February 2020, reached record highs.

**Figure 2:**
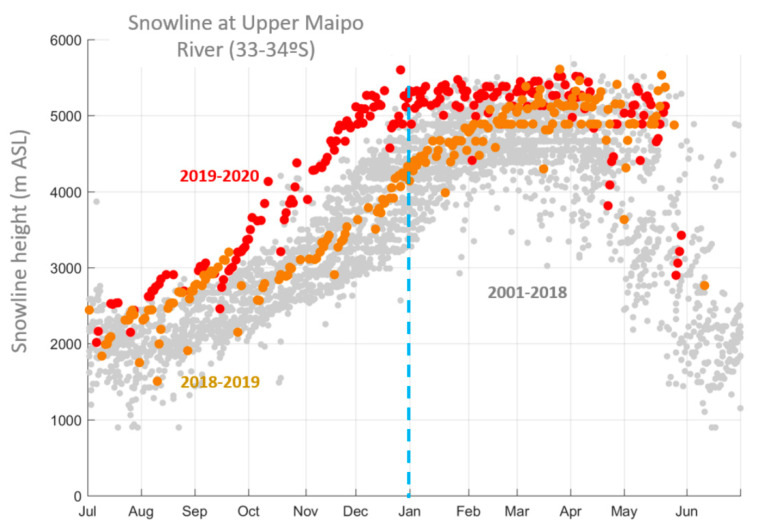
Annual cycle and interannual variability of the snow-line in central Chile (upper Maipo river basin) close to our study area over the past decades (grey circles). Each circle is a given day in a year. The period July 2018–June 2019 is highlighted in orange and the period July 2019–June 2020 (extreme drought) is highlighted in red.

Both the very low precipitation during the 2019 winter (affecting the late summer months of the austral summer of 2019–2020 at higher elevations) and the warm anomalies during spring and summer, moreover, resulted in unprecedented low snow cover over central Chile, particularly in the latitudinal range corresponding to our study area. Figure 2 shows the daily elevation of the snow-line (the lower limit of the extent of snow, as detected by satellites) for each hydrological year from 2000 to 2020. The last two years are highlighted: while June 2018–June 2019 values were near normal, for the period July 2019–June 2020, the snow-line was the highest on record. The largest absolute difference occurred in October–November when the 2019 snow-line lay close to 1500 m elevation above its average position for the period covered by the data source. Recalling that the crest of the Andes in the region lies at about 5500 m.a.s.l. (to the west), Figure 2 reveals that the entire seasonal snow pack was fully gone by early December 2019, about 2 months earlier than in a normal year. This rapid reduction in the Andean snowpack probably translated into the absence of ephemeral springs and groundwater. The latter, together with scarce snow in the winter months, would have been obstacles for the resumption of plant growth after snowmelt in the semi-arid central Chilean subalpine landscape.

### 2.2. Onsite Weather Conditions over the Study Period

**Figure 3:**
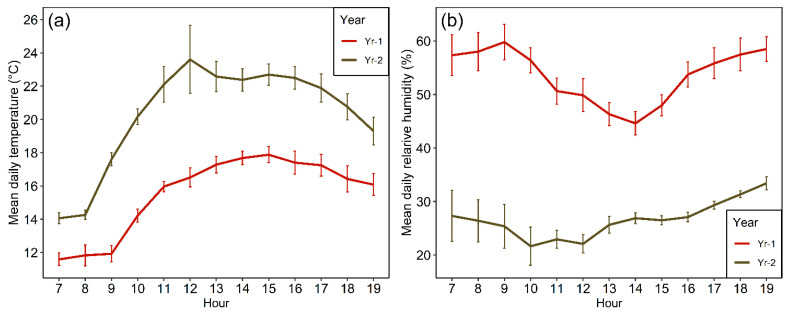
Average (+1SE) hourly daytime temperature (**a**) and relative humidity (**b**) between 7 h (7 a.m.) and 19 h (7 p.m.) standard time over the study periods in February 2019 (Yr-1) and 2020 (Yr-2) based on the authors’ onsite recordings.

The detailed work on the impacts of drought on pollination, nectar and seed set was carried out on a high elevation site (2450–2500 m.a.s.l.) in the Andes located some 36 km in a straight line east of Santiago in February of 2019 and 2020, respectively (hereafter Yr-1 and Yr-2). Reflecting the subalpine climate, hourly onsite recordings made by us indicate that the mean monthly temperature for September through February was 10.6 °C in Yr-1 and 11.5 °C in Yr-2. The warmer conditions in the 2019–2020 austral spring–summer period are consistent with the previous discussion based on Santiago records. The higher air temperatures in Yr-2, further, were associated with a reduction in relative humidity decreasing from 52.3% in Yr-1 to 43.7% in Yr-2. Overall, daytime temperature and relative humidity (Figure 3) were noticeably different for the two years during the specific dates of the pollinator sampling (middle of the second week of February in 2019 and 2020 onwards, respectively). In the regionally much drier Yr-2, temperatures tended to be higher for all hours of the day while relative humidity was lower (Figure 3). Differences in hourly temperature and relative humidity between years averaged 4.8 °C (range: 2.4–7.1 °C) and 27% (range: 17.1–34.8%), respectively. All daytime hours were cloudless in 2020 whereas in 2019 only 53% of the sampling hours were completely cloudless. Daytime hourly wind speed averaged 5.90 kph and 5.89 kph in 2019 and 2020, respectively, and was not significantly different between years (t_106_ = 0.099, *p* = 0.921). Overall, recorded wind speed failed to exceed 12 kph in both years. No rain was received during the pollination observation days in either year.

### 2.3. Selfing Capacity, Flower-Head and Individual Floret Longevity

**Figure 4:**
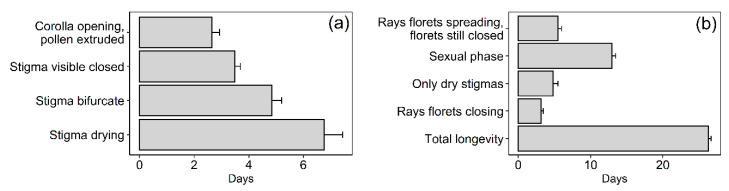
Potential floral longevity in *M. subulata*. (**a**) Duration of the male, neutral (stigmas emerging, not bifurcate), receptive stigma and dry stigma stages of individual florets (Mean + SE, N = 25). (**b**) Total longevity of pollinator-excluded flowers-heads (Mean + SE, N = 15) showing the duration of different stages.

A mere 2.3% of the total florets contained in the pollinator-excluded flower-heads of *M. subulata* set seed, indicating a clear lack of autonomous selfing capacity. This very small amount of seed was concentrated in two of the total of 15 heads that could be retrieved (from 15 plants). Further evidence for allogamy was obtained by monitoring the developmental phases and longevity of individual florets. Individual florets of pollinator-excluded heads remained open for an average of 17.7 d (Figure 4a). Pollen was actively extruded for 2.6 d after which time the stigma elongated over a period of 3.5 d to become bifurcate and then receptive for a period of 4.8 days, indicating strong protandry. Thereafter, the stigma became dry and remained in that state for almost one week.

In Yr-1, onsite pollinator-excluded flower-heads remained open for an average of 26.3 d, with the sexual phase (pollen presentation to presence of turgid bifurcate stigmas) lasting 12.9 d (Figure 4b). Comparison of the length of the overall sexual phase of the flower-head (Figure 4b) with that of the individual florets (12.9 vs. 10.9 d) indicates the many florets are open synchronously. Interestingly, the functional sexual phase of the head, although fairly long, was relatively short in comparison with total head longevity (49% of total duration). In the warmer and drier Yr-2, a single flower-head that could be successfully time-lapse filmed remained open for only 18 days, which is considerably shorter than the minimum of 24 d observed in Yr-1, suggesting that more severe drought affected potential flower-head longevity.

### 2.4. Flower Traits and Pollinators

**Figure 5:**
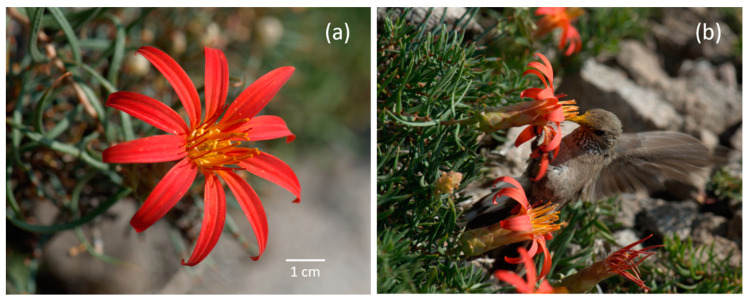
(**a**) Typical flower-head of *M. subulata* fma. *rosmariniifolia* (Asteraceae) showing the brilliant red ray florets, disk florets with golden-yellow corollas, dark red elongate stigmas and abundant yellow-orange pollen being extruded by the florets. (**b**) *Oreotrochilus leucopleurus* (White-sided Hillstar hummingbird—Picaflor de Cordillera) visiting an exceptionally dense patch of *M. subulata* on the study site. The stems of *M. subulata* are growing between the branches of short-statured shrubs of *Ephedra chilensis.* Images: Mary T. K. Arroyo.

The large red flower heads of the subalpine form of *M. subulata* (Figure 5a) are mostly disposed close to the ground and are oriented at around 45° with respect to ground level (Figure 5b). This orientation comes about on account of conspicuous angling of the supporting peduncle just below the receptacle bringing the heads into the typical position seen in Figure 5b.

In the two years we accumulated a total of 19,440 min of pollinator observations (9 d × 12 h × 45 min × 4 observers). As expected from its floral syndrome, hummingbirds were by far the most important visitors to *M. subulata* in both years, overall accounting for 99.7% of total visits. All hummingbird visits (Table 1) pertained to the single large, high-elevation hummingbird species, *Oreotrochilus leucopleurus* Gould (Figure 5b). Individuals of this species visited *M. subulata* throughout the day from prior to 7 a.m. to after 7 p.m. Upon visiting, the hummingbirds’ heads become covered with the yellow-orange pollen (Figure 5b). The birds typically secured the low-growing flower-heads with their claws either via their peduncles or ray florets while using their tails to balance on the ground (see Figure 5b, Videos S1 and S2). When the flower-heads were situated very close to the ground, or trailed over rocks, they stood on the ground or on the rocks while probing the florets. Visits in suspended flight were rare. Abundant butterfly flight bouts (mainly by *Etcheverrius chiliensis*, *Auca coctei*, *Faunula leucoglene*) were also recorded in the observation plots (Table 1). However, butterflies rarely alighted on the flower-heads or probed florets in either study years. The only other flower visitor was a bumblebee (*Bombus dahlbomii*) which made a total of three visits in 2019. Notably, the hummingbirds also flew into the plots many times without making visits (Table 1). Such activity was associated with territorial behaviour. Examination of untreated pollen grains (N = 454) scraped off a captured hummingbird’s head revealed that 95.6% of the large, elongated, strongly sculptured grains belonged to *M. subulata*, as per comparison with published images of *Mutisia* spp. grains [48]. The remaining gains (except one) were either *M. subulata* as seen from a polar view or an unidentified species.

**Figure 6:**
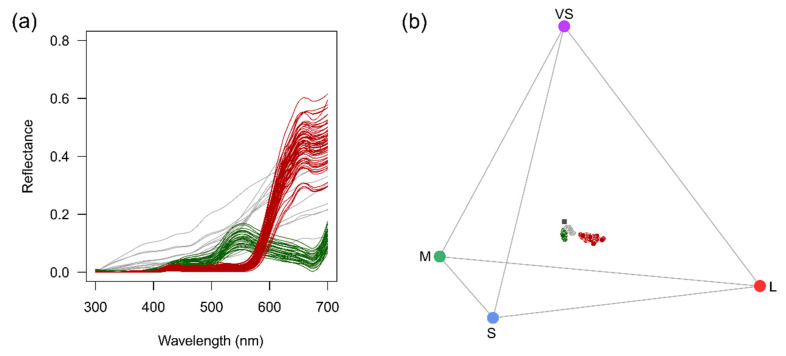
(**a**) Spectral reflectance of ray florets and leaves of *M. subulata* and of stones against which flower-heads are likely to be detected by pollinators given the low disposition of the flower-heads. (**b**) Distribution of ray floret, leaf and stone colours in the avian tetrahedral space considering the standard avian colour vision system with VS, S, M and L type cones which correspond to cones maximally sensitive to violet, blue, green and light red, respectively. Lines and symbols: red = ray florets; green = foliage; grey = stones.

Further evidence, consistent with *M. subulata´s* adaptation to hummingbird pollination, was forthcoming from the reflectance spectra of the showy rays florets. The latter showed low reflectance between 300 and 580 nm and high reflectance at wavelengths above 600 nm (Figure 6a), accounting for their typical red appearance, as shown in Figure 5a. Although these wavelengths do not exclude other pollinator groups, they are typical of bird-pollinated species.

Given the low height above ground at which *O. leucopleurus* is obliged to forage, we also obtained spectral measurements for leaves and stones to look for contrasts. Leaves, not surprisingly, showed the typical plant foliage spectra having enhanced green reflectance between 500 and 600 nm, and moderate to low reflectance in the other parts of the visible spectrum (Figure 6a). In the case of stones, although variation was found, the spectra were characterised by a steady increase in reflectance towards longer wavelengths, which in some cases was more appreciable above 550 nm and accounted for their dark orange appearance (Figure 6a). Distribution of colours in the avian tetrahedral space shows that the ray florets occupy loci well separated from the loci occupied by leaves and stones (Figure 6b).

### 2.5. Interannual Variation in Flower-Head Abundance and Visitation

The exceedingly severe drought in Yr-2 was expected to reduce floral resources given that more plants were seen to be dry that year. We documented a huge difference in flower-head density. Total flower-heads open per day in the four 15 × 15 m pollinator observation plots varied from 459 to 533 in Yr-1, as compared with 28 to 51 in Yr-2. The mean number of open flower-heads per plot considering the data for all sampling days was significantly higher in Yr-1 (Mann–Whitney U-test, U = 320, *p* = 3.74 × 10^−7^, Yr-1, N = 20; Yr-2, N = 16). Taking into account the mean number of flower-heads open per day in the four sampling plots (900 m^2^) each year, there were 92% fewer flower-heads in Yr-2.

**Figure 7:**
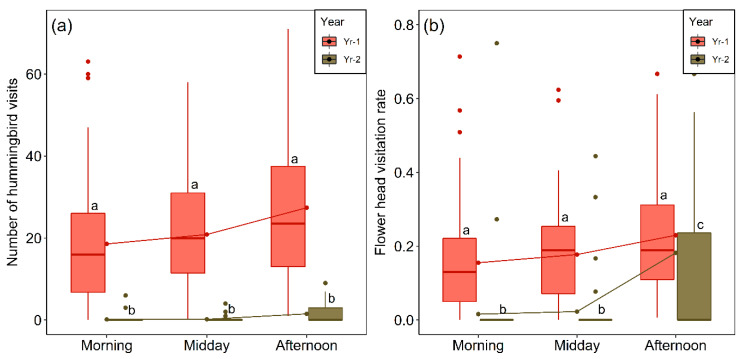
(**a**) Comparison of number of visits to flower-heads of *M. subulata* in equal-sized sampling areas (visitation frequency) over different periods of the day for the last two years of the Chilean mega drought. The raw data are the number of visits per hour per plot on each day of pollinator sampling (Yr-1, N = 240; Yr-2, N = 192). (**b**) Flower-head visitation rates per hour per plot in *M. subulata* over different periods of the day for same two years. Outliers (values higher than 1.5 times the interquartile range above the upper quartile) are shown as dots. Lower case letters indicate significant differences between years (*p* < 0.05) according to Dunn’s test.

Both year and period of the day and their interaction had highly significant effects on the frequency of visits (Table 2). Although we found an overall difference in the number of visits among the three periods of the day (χ^2^ = 10.107, *p* = 0.0064, Kruskal–Wallis test), for the individual years such differences were not statistically significant (Figure 7a). Overall the number of visits per day in Yr-1 was 36.5 times higher than in the much drier and warmer Yr-2. The percentage of total flights (which can be with or without visits) into the sampling areas that involved visits was higher in Yr-2 (80%) than in Yr-1 (52.5%), indicating that the birds dedicated relatively more of their time to collecting nectar in Yr-2. When we separated the sample of 168 flowering heads mentioned in Section 4.1 according to year (Yr-1, N = 100, Yr-2, N = 68), flower-head diameter and disk floret number were not significantly different among years (flower-head diameter: Mann–Whitney U-test, U = 3167.5, *p* = 0.45; disk floret number, Mann–Whitney U-test, U = 3850, *p* = 0.145), although for the same flower-heads, rays floret number was larger in Yr-2 (Mann–Whitney U-test, U = 2478.5, *p* = 0.002). Thus, differences in visitation frequency cannot be put down to larger and more attractive flower-heads in Yr-1. We recorded between one and—exceptionally—36 probes, on a single flower-head visit. However, the number of probes per flower-head was significantly lower in Yr-2 (median three in Yr-2 versus five in Yr-1; Mann–Whitney U-test, U = 28436, *p* = 0.0248, Yr-1, N = 551; Yr-2, N = 48).

Year and period of the day also had highly significant effects on flower-head visitation rates, calculated as the mean number of visits per flower-head (Table 2, Figure 7b). Differences between the three periods of the day were not significant in Yr-1 but in two cases in Yr-2 they were (afternoon vs. morning, *p* = 0.0001 and midday vs. afternoon, *p* = 0.001, Dunn´s test). Reflecting the results for visitation frequency (Figure 7a), all pairwise differences for the same period of the day between years were significant (Dunn´s test, *p* < 0.0001 in all cases) (Figure 7b).

### 2.6. Interannual and Diurnal Variation in Nectar Production and Availability

**Figure 8:**
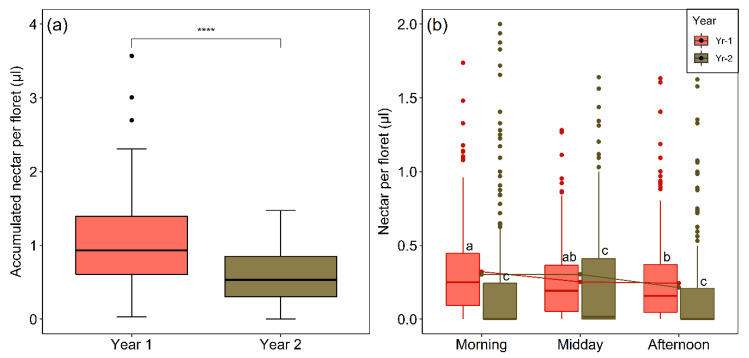
(**a**) Amount of nectar accumulated in individual florets in pollinator-excluded flower-heads of *M. subulata* over the last two years of the Chilean mega drought (Yr-1, N = 88; Yr-2, N = 77). The four asterisks (****) indicate a significant difference between years (*p* < 0.0001) based on the Mann–Whitney U-test. (**b**) Nectar standing crop per floret for the two years over different periods of the day (Yr-1, N = 735; Yr-2, N = 570). Outliers (values higher than 1.5 times the interquartile range above the upper quartile) are shown as dots. Four outliers were excluded from (**b**) to facilitate visual presentation. Note that these outliers were not excluded from the statistical analysis. Lower case letters indicate significant differences (*p* < 0.05) according to Dunn’s test.

Accumulated nectar per floret in pollinator-excluded flower-heads generally failed to exceed 1 µL (Figure 8a). However, considering the mean of 20.5 long-lived disc florets in a flower-head (see Section 4.1) and which were shown to open fairly simultaneously and seen to continue to have nectar after the stigmas fail to be receptive, the flower-heads of *M. subulata* are a rich source of nectar. Significantly less nectar accumulated per floret in the pollinator-excluded heads in Yr-2 (Figure 8a). Nectar standing crop volume per floret was much smaller and more variable than total accumulated nectar per floret (c.f., Figure 8a,b). The effect of both year and period of the day and their interaction on nectar standing crop were highly significant (Table 3, Figure 8b), with nectar volume always being larger in Yr-1 when equivalent periods of the day are compared (*p* < 0.0001 in all cases, Dunn´s test). In Yr-2, where the nectar volumes were smaller, there were no significant differences over the day. In Yr-1, florets extracted in the afternoon had less nectar than in those extracted in the morning (*p* < 0.025, Dunn´s test). The lower nectar standing crop in Yr-2 was strongly influenced by a much higher percentage of florets lacking extractable nectar (Yr-1, 8.4% Yr-2, 52.6%).

Nectar concentration obtained from other flowers harvested over different periods of the day in Yr-1 averaged 35.5% (N = 32 flower-heads, SE = 1.3). The Yr-2 samples gave very high concentrations ranging from 66.7 to 78.3%. Although we had far less data for Yr-2 (because of a lack of available flower-heads) the results clearly show that the nectar was far more concentrated that year.

### 2.7. Seed Set

**Figure 9:**
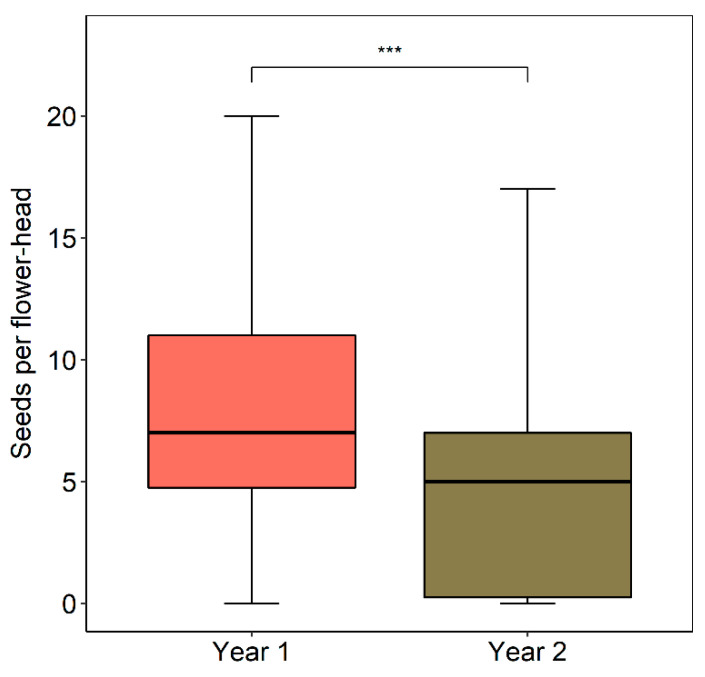
Seed set per flower-head in *M. subulata* over the last two years of the Chilean mega drought. Yr-1 is the austral summer of 2018–2019; Yr-2 is the austral summer of 2019–2020. Retrieved flower-head sample sizes were N = 132 (Yr-1), N = 66 (Yr-2). The three asterisks (***) indicate a significant difference between years (*p* < 0.001) based on the Mann–Whitney U-test.

Seed (achene) set per flower-head was significantly higher in Yr-1 (Figure 9). In relative terms, the absolute number of seeds per flower-head in Yr-2 was 31% lower compared to Yr-2. In terms of seeds per ovule, seed set dropped from an average of 36.2 to 26.7%. At a smaller spatial scale, as was suspected, seed set per flower and the seed/ovule ratio tended to increase with increasing floral density as per in the 15 × 15 m observation plots, although in both cases the tendencies failed to be significant (seed set per flower-head, R = 0.460, *p* = 0.252; seeds per ovule per flower-head, r = 0.444, *p* = 0.271, d.f. = 6 in both cases). The lower seed set in Yr-2 is partially due to a higher percentage of the heads that year not having any seeds at all (Yr-1 = 6.8%, Yr-2 = 25.8%, χ^2^ = 13.8366, *p* < 0.001). Considering only the flower-heads that had some seeds, the seed set in Yr-1 heads was marginally higher than in Yr-2 (Mann–Whitney U Test, U = 2498, *p* = 0.080, Yr-1, N = 123, Yr-2, N = 49).

### 2.8. Effect of Direct and Indirect Drivers on Pollinator Activity

In an effort to sort out which factors best explain the variation in pollinator visitation seen over the two years we conducted linear and generalised linear mixed models using different combinations of the following variables: cloud cover, temperature, wind speed, relative humidity, nectar standing crop, flower-head availability (see Table S1 for original data). The best model for plot-level visitation frequency according to the Akaike information criterion (AIC = 2982.903) was a linear model that included nectar standing crop, temperature, relative humidity and flower-head availability as predictor variables. Neither wind-speed nor cloud cover featured in the model. The selected model variables were not collinear (flower-head number, VIF = 3.38, relative humidity, VIF = 4.47; nectar standing crop, VIF = 1.13; temperature, VIF = 2.32).

Flower-head density, relative humidity and temperature all had significant and positive effects on visitation frequency (Table 4). The effects of flower-head density and relative humidity were clearly the strongest. Surprisingly, nectar standing crop, which was significantly reduced in Yr-2, had no significant effect on visitation frequency. Interestingly, although higher temperatures had a positive effect on visitation frequency in the subalpine location, that effect was insufficient to override the negative effects of lower relative humidity and lower flower-head density.

## 3. Discussion

The year 2019, leading up to the 2019–2020 austral summer, was an extreme drought year in central Chile with precipitation deficits in the 70–90% range in relation to the longer-term mean across the region, comparable only to 1924, 1968 and 1998. Moreover, that year culminated a decade-long mega drought brought on by a combination of natural variability and anthropogenic forcing [2,49]. The spring (2019) and summer (2020) were the warmest since 1960, reflecting long term warming in the interior valleys and the Andes of north-central Chile [50] seemingly connected with global climate change [51]. Such extreme warm and dry conditions resulted in an unprecedented short seasonal snow pack and depleted surface and groundwater which would have reduced soil moisture at all elevations in the subalpine and high alpine zones. Examination of meteorological data for Santiago reveals a tendency, albeit weak, for larger precipitation deficits to be accompanied by higher spring and early summer temperatures (see Figure A2). One may speculate that such a correlation emerges from some local effect. One possibility is that less precipitation during winter reduces soil moisture, lessens latent heat flux and increases sensible heat flux, thus resulting in warmer conditions in the following spring and summer. Nevertheless, the trend, in part, is due to concurrent, multi-decadal drying and warming. A comparison of the last two summers of the central Chilean mega drought provided a unique opportunity to assess the effect of a very severe drought year in combination with warmer weather on a key biotic process.

The subalpine form of *M. subulata*, our focal species, was shown to possess strongly protandrous disk florets and lack autonomous selfing capacity. Flower-head colour and morphology confirmed a hummingbird-pollination syndrome which was amply verified by abundant visitation by the high-elevation hummingbird, *O. leucopleurus*. The low-disposed flower-heads are particularly interesting. They were seen to be associated with unusual foraging habits, previously described for the same hummingbird on *Caiophora coronata* [52] and other high elevation Andean hummingbirds [53]. Due to the fact that we studied a single population of *M. subulata* on a fairly cold slope, we cannot discard the possibility that *Patagona gigas,* another hummingbird found in the same general area (but which is more common at lower elevations), could eventually turn up at warmer sites with *M. subulata*. Nevertheless, it is telling that *O. leucopleurus* was the only hummingbird seen on *M. subulata* at two additional subalpine locations north and south of our study site separated by some 170 km. Overall, these results, taken together, lead to the conclusion that the subalpine form of *M. subulata* is a strongly pollinator-dependent and highly specialised hummingbird-pollinated plant. Overall, we recorded very few butterfly visits on our site. However, more butterfly pollination perhaps occurs when flower-head density is low at the beginning and end of the flowering season or when populations of *M. subulata* are very small and would be unattractive to a high-energy demanding pollinator. Visits by butterflies to red hummingbird-pollinated flowers are not unexpected as butterflies typically have a colour vision system that includes photoreceptors maximally sensitive to red [54].

We found significant reductions in flower-head abundance, visitation frequency, flower-head visitation rates, nectar standing crop and seed set in the driest summer. Trends of these kinds have been reported in experimental studies including different watering treatments [16,18,55]. For seed set, indeed, the seed/ovule ratio was already on the low side in the first year compared with that reported for perennial plant species in general [56]. Aborted seeds were seen in both years. It is always possible that there was more seed abortion in the drier year because of a lack of plant resources, including water. Indeed, a recent study using different soil moisture treatments detected decoupling of visitation rates and seed set, which appeared to be due to an independent effect of soil moisture on seed set to the extent that final seed set was perhaps not always strongly pollinator-limited [16]. The fact that significantly more flower-heads lacked good seed in Yr-2 in our species would tend to argue against this, although it must be recognised that the latter result could have been affected by very early seed abortion in the Yr-2 flower-heads. Distinguishing between very early seed abortion and unfertilised ovules is very difficult in Asteraceae. In any case, summing together the fewer available ovules due to the reduced number of flower-heads and the lower seed set, the overall reduction in seed production on the site, in what was the severest year of a 10 year drought, clearly would have been substantial.

We were particularly interested in investigating the role of direct and indirect climatic drivers on pollinator visitation which barring some recent exceptions, have tended to be considered separately. Our models for *M. subulata* showed flower-head density and relative humidity to be the most important drivers. Lower flower-head density and lower relative humidity (to be discussed later) were both associated with fewer visits, as dramatically seen in Yr-2, when it was also seen that flower visitation rates decreased. In the only previous study that, to our knowledge, has looked at the effect of drought in a hummingbird-pollinated plants, flower visitation rates increased [34]. According to the authors, the resident birds were forced to visit a larger number of flowers in order to obtain sufficient nectar. In our case, although the nectar standing crop in *M. subulata* was significantly reduced in the driest year and therefore suggestive, it cannot be presumed the cause of reduced visitation, given that no significant effect of nectar standing crop emerged in the best model to explain visitation frequency. One possibility to explain our results is that the very low flower-head density in the driest year led *O. leucopleurus* (given the acute spatial memory of hummingbirds) to perceive an energetically precarious nectar resource and consequently lowered its density substantially, leading, in turn, to lower flower-head visitation rates. It is well known that hummingbirds are able to assess the profitability of floral patches and associate floral attributes such as flower colour with potential energetic rewards [57,58,59].

Significant hummingbird mortality has been documented in drought years in other ecosystems [33]. It is therefore plausible that the lower visitation frequencies and, counterintuitively, lower flower-head visitation rates, in the driest year were due to bird mortality in *O. leucopleurus* stemming from an inability to collect sufficient resources to maintain a positive energy balance. A third possibility is that *M. subulata´s* single pollinator spread its nectar foraging over more plant species. The feeding ranges of individual hummingbird species are known to be inversely proportional to flower density [60]. Moreover, seasonal decreases in plant species richness can provoke increased overlap in the nectar diet of hummingbirds [61]. Northern Andean species of *Oreotrochilus* are known to concentrate on a particular plant species at any one time [62]. Although long-term studies have found high site fidelity among years in some hummingbird species [63], under extreme drought, it would not be surprising if some individuals of *O. leucopleurus* had extended their feeding ranges to include other plant species less affected by the drought. In this vein, in late February 2020, we observed *O. leucopleurus* foraging actively on *S. hookeri*, a generalist plant species pollinated by bees and hummingbirds [64,65], which that year appeared in the then dry water courses on the same slopes occupied by our broader population of *M. subulata*. However, whether these were the same individuals that visited *M. subulata* is unclear. If *O. leucopleurus* was able to increase its foraging range to include more species, clearly, among the two mutualists, it was less at risk than specialised *M. subulata.* To fully understand the consequences of extreme drought and global warming on hummingbird-pollination systems, a pollination network approach considering different spatial scales, combined with bird banding or radio tracking, is desirable.

We found a positive effect of ambient temperature on hummingbird visitation. Higher temperatures associated with global warming could disrupt plant–pollinator interactions either through altering pollinator emergence dates or exceeding optimal temperatures for pollinator foraging [30,66,67]. While warmer temperatures favoured hummingbird activity on the subalpine site, an earlier community-level study in the same general area, in which bee- and fly-pollinated species were strongly represented, showed flower visitation rates in the subalpine to decline when temperatures around flower height exceeded the 20–25 °C range [22]. In the upper alpine, the critical upper temperature range was around 15–20 °C. In the second year of our study, hourly daytime temperatures exceeded 24 °C seven times in a single day (rising to over 29 °C at one point) and were over 20 °C on several occasions on the remaining days. As temperatures close to the ground can be higher than the air temperature in the central Chile Andes [68], it is likely that the critical temperatures for bee and fly activity were reached frequently in the general area in Yr-2. In any case, determining the thermal niches and where the upper temperature bounds lie for different pollinator groups in different ecosystems are critical questions. Our findings in *M. subulata*, together with the earlier work alluded to, tell us that the responses of pollinators to climate change are likely to vary not only according to pollinator group but also in relation to ecological context.

We were surprised to find nectar standing crop volume, although significantly reduced in the driest year and selected in the best model, was not a significant driver of visitation frequency. A similar result was found in a recent study in a bumblebee-pollinated plant [16]. Perhaps if we had been able to include sugar concentration in the models, a significant nectar effect would have emerged. Unfortunately, for technical reasons, it was impossible to measure sugar concentration in the field over the pollinator sampling days. Our results for relative humidity could provide an important clue regarding the role of nectar. Low relative humidity is well known to lead to more concentrated nectar [41]. Therefore, the model effect of this climatic variable likely reflects nectar concentration. We hypothesize that the Yr-2 nectar (which was shown to be very concentrated based on other samples analysed in the laboratory), in exceeding the optimal concentration of 30–40% for capillary suction feeders, as are hummingbirds [69], lowered flower-head visitation via a learning experience as per discussed earlier. This argument gathers support from the much higher percentage of florets lacking extractable nectar in the drier and warmer year compared with the previous year which was cooler and had a higher relative humidity. Empty flowers have been reported for other plant species under drought conditions [42,43]. Finally, the huge reduction in extractable nectar we saw in Yr-2 cannot be put down to the hummingbirds removing greater volumes of nectar, first because there were fewer visits per flower-head, and second, the birds probed fewer florets per flower-head visited. We suggest nectar concentration is likely to be the reason behind the fewer florets probed per flower-head visit, and ultimately, part of the explanation for the overall lower flower-head visitation rates.

It is worth delving more deeply into why there were so fewer flower-heads on our site in the driest year. Mass flowering in what appears to be a long-lived subshrub, whereby fewer flower-heads are produced following a year of very abundant flowering, is always a possibility, but impossible to evaluate without longer-term studies. As mentioned earlier, there were some indications of florivory, but whether florivory is greater in drier years needs further study. The most obvious explanation is water stress affecting plant growth and resource allocation, alluded to earlier. Interestingly, while hummingbird visitation to *M. subulata* benefited from warmer temperatures, flower-head density would have suffered because the warmer temperatures in the second year (possibly in part tied to the huge precipitation deficits in 2019, as discussed above), if anything, would have exacerbated soil drying over that produced by the large precipitation deficit, thereby further reducing plant growth and flowering. Separating the indirect and direct effects of the longer-term warming in the Andes of central Chile from those produced by other sources of temperature variation would require several years of data and thus was beyond the scope of the present study.

Our results suggest long-lived flower-heads could also play a role in explaining the reduced floral abundance in Yr-2. Although several were lost, Yr-2 flower-heads seemed to last fewer days. This is consistent with other studies in the Chilean Andes which show that single flowers stay open longer upon water addition [70], in wetter years [45] and under cooler temperatures [22]. Shorter lived flower-heads imply less temporal overlap among open flower-heads and hence fewer flower-heads open at any one time. The take-home message is that drought can affect floral abundance directly through vegetative growth and indirectly through its effects on floral longevity.

Given that the snow disappeared earlier under the warmer spring of the severest year of the drought, conceivably the lower flower-head abundance that year was conditioned by much earlier flowering. The latter can be reasonably discarded on three counts. First, we found few open flower-heads 11 days before the pollination survey began in February 2020. Second, as in 2019, we saw few fruiting heads of *M. subulata* at the start of the pollinator sampling. Third, we know that flowering continued in 2020 at least into late February. Interestingly, high water stress associated with drought is known to delay flowering [38,71,72]. In this respect, significantly, the subalpine belt in the dry central Chilean Andes normally suffers water stress as the summer proceeds [73]. It is well known that warmer global temperatures are advancing flowering worldwide [74,75,76,77,78]. Under the current long-term trends of decreasing precipitation in combination with increasing temperatures in central Chile [12,50,79], we predict that advanced flowering is less likely in the central Chilean Andes.

Returning to the first objective of this paper, the basis of pollination specialization in *M. subulata* is worthy of further comment. We currently have no explanation for the lack of butterfly visits, except possibly that the butterflies were in some way discouraged by the hummingbirds, and/or in both years temperatures close to the ground were too warm for them. While the general lack of bee visits is consistent with the type of spectral reflectance measured in *M. subulata* [80,81,82], the virtual absence of visits by the bumblebee *B. dahlbomii*, given some unusual characteristics it has, was unexpected. It could have something to do with the fact that the flower-heads of *M. subulata* are usually disposed close to the ground. Despite the fact that its colour vision lacks photoreceptors maximally sensitive to red, *B. dahlbomii* is a common visitor to red flowers in southern South America [83,84]. It has been proposed that when lying over a green foliage background, as in forests, red flowers will appear as dark objects over a bright background due to an achromatic mechanism mediated by the bumblebee´s green photoreceptors [83]. In our study area, the flower-heads of *M. subulata* were frequently found against a background of stones whose spectral properties corresponded to dark orange and thus would have tended to hinder the red flower-heads being detected by the bumblebee. We hypothesize that the low detectability of flower-heads for bumblebees seen on our study site acts synergistically with other floral traits and environmental factors leading to few visits by the native bumblebee [85]. In contrast, the tetrachromatic colour vision of hummingbirds with cone type photoreceptors sensitive to violet, blue, green and red light [86,87,88] allows them to detect the flower-heads of *M. subulata* irrespective of the background over which they are encountered. In summary, a combination of biotic and abiotic factors determines the specialised pollination in the subalpine form of *M. subulata.* As a caveat, it would not be surprising if the taller lower-elevation matorral form of our species and forest-dwelling species of the genus in southern South America, where the flower-heads are commonly found over a background of green foliage, turn out to be visited by both hummingbirds and *B. dahlbomii*.

Finally, hummingbirds are known to visit many other species of South American *Mutisia* in a plant family in which ornithophily is exceedingly rare [89,90,91,92,93]. Along with the new information for *M. subulata* fma. *rosmarinifolia* presented on this occasion, we report visits by the lower-elevation hummingbird, *Sephanoides galeritus* on *M. ilicifolia* with large, dark pink, flower-heads. This same hummingbird is likely to pollinate the montane and sclerophyllous forest form of *M. subulata* (fma. *subulata*). Given the diversity of climates it occupies [94], the genus *Mutisia* offers an outstanding opportunity for furthering our understanding on how climate change and climate anomalies affect hummingbird-pollinated species.

## 4. Materials and Methods

### 4.1. Focal Species

M. *subulata* Ruiz and Pav. fma. *rosmarinifolia* (Poepp. and Endl.) Cabrera, the subalpine form of the species, is restricted to the central Chilean Andes where it occurs from ca. 30° S to 37° S, and between 1700 and 3000 m.a.s.l [95] depending on latitude, with a preference for well-drained slopes with moderate-deep winter snow accumulation. *M. subulata* fma. *rosamarinfolia* is a low-growing subshrub characterised by horizontal stems. The leaves lack well-developed tendrils. In contrast, the lower-elevation fma. *subulata* which is found mostly below treeline to around 1000 m.a.s.l., is a strongly climbing subshrub. As in fma. *subulata*, the branches of the subalpine form have large brilliant red terminal flower-heads. However, while the flower-heads of fma. *subulata* are found high up among the branches upon which the plant twines, those of the subalpine form are mostly disposed close to the ground (15–20 cm a.g.l.). The number of flower-heads varies according to the number of branches on a plant which are generally few, although clumps of flower-heads can occasionally be found when individual plants are matted together. The highest flower-head density recorded on our plots in any one day over the two years of our study was less than one flower-head per m^2^. The flower-heads measure 6.3 + 0.11 cm in diameter (measured in the field) and have 9.9 + 0.11 ray and 20.5 + 0.35 disk florets (mean + 1 SE, all measurements on N = 168 heads, one per plant). The individual disk florets are large and slightly zygomorphic and contain a large disk-like nectary located at the top of the ovary, 2 mm height. The corolla tube and its flare together measure 2.6 ± 0.01 cm (Mean ± SE, N = 300, 3 disk florets per each of 100 flower-heads). The style is very long and strongly exserted (see Figure 5a). We confirmed that nectar secretion is lacking while the florets are still closed. For a sample of 62 unopened florets (N = 11 plants), 71% were totally empty with the remainder showing slight dampness. As is typical in Asteraceae, pollen (bright yellow-orange) is pumped out of the opening florets by the elongation of the style whose stigma later bifurcates to become receptive. The ray florets are sterile. Time lapse videos made by us confirmed that the ray florets show diurnal movement whereby the rays are more spread in the middle of the day. The heads fail to close at night.

Flowering in *M*. *subulata* fma. *rosmarinifolia* (which has been referred to as *M. subulata* throughout the paper for simplicity), mirroring that in many subalpine species of Asteraceae in the central Chilean Andes [96], occurs fairly late in the summer season (end of January–March on the specific site considered). At this time of the year, soils are compact and very dry. Other plant species still in flower in February and/or March in the subalpine belt in the central Chilean Andes reported to be amply pollinated by hummingbirds are *Schizanthus hookeri* and *S. grahamii* (Solanaceae) [64,65] and *Eythranthe lutea* (formerly *Mimulus luteus*) (Phrymaceae) [97]. While none of these species were found in our sampling plots, they all occur on the same slopes and in one case (*S. hookeri*) occurred within a few meters of plants of *M. subulata* further along the slope.

### 4.2. Study Site

**Figure 10:**
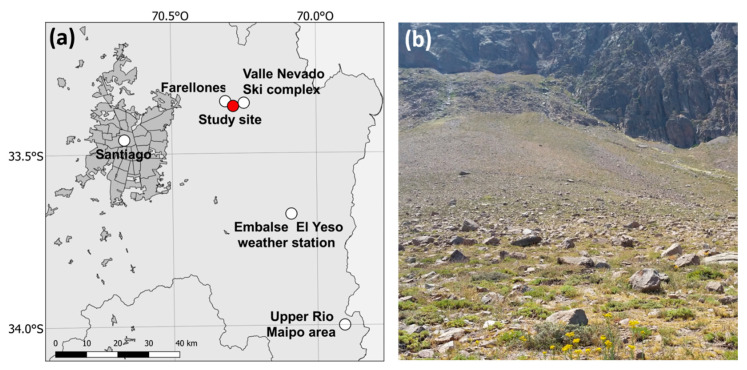
(**a**) Location the study site (red filled circle) at 2400–2450 m.a.s.l. in the Andes east of the city of Santiago, Metropolitan Region, Chile. Also shown are other localities mentioned in the text. (**b**) View of the subalpine slopes where the work was carried out. Image: Mary T. K. Arroyo, taken in Yr-1 on 21 February 2019.

The exact site is found on a southwest-facing slope above the road between Farellones and Valle Nevado, Metropolitan Region of Chile, 2400–2500 m.a.s.l (33.3628° S, 70.2870° W) (Figure 10a). The area is characterised by a subalpine climate with a strong influence of the typical Mediterranean-type climate that characterizes subtending lowland areas [96]. During the summer months (November–March), the strong influence of the SE Pacific subtropical anticyclone leads to very dry, warm and stable conditions over central Chile. Except for some sporadic convective activity atop of the Andes, the summer months receive sporadic showers or no rain at all [98]. Annual average precipitation in the general area at 2500 m.a.s.l. is estimated to be c. 445 mm. Mean annual temperature for the Embalse del Yeso Station to the south (33.6767° S, 70.0886° W, 2475 m.a.s.l.) (CR2 Climate Explorer available at http://explorador.cr2.cl/, accessed 30 September 2020, data from 1977 onwards) is 8.7 °C. Examination of MODIS/Terra sensor data [99] showed that the snow lifted definitively around 26 September in Yr-1 and 17 September in Yr-2. The work reported here was carried out as of the middle of the second week of February onwards in 2019 and 2020, respectively. Based on reconnaissance work at the end of January in Yr-1 these dates corresponded roughly to the peak onwards of the flowering season.

The population of *M. subulata* studied extends in a band along a mountainous slope for around 0.85 km varying widely in plant density. Occasional cows and horses were seen on the slopes in both years but did not seem to favour the thick stiff leaves of *Mutisia*. We selected the middle part of the population (Figure 10b) where *M. subulata* was abundant and easily accessible. In the first year, the study area was subdivided into two similar-sized areas, a flatter lower area where we undertook harvesting for nectar extraction, extracted plants for floral measurements and conducted our observations on flower-longevity and the capacity for pollinator-excluded set seed. The upper, somewhat steeper, rockier and better-drained part of the population was reserved mainly for observations on pollinators and open-pollination seed set. In dividing the work space this way we sought to reduce interference and minimize extraction in the upper area during the pollinator observations. The areas sampled for nectar were contiguous with the pollinator sampling area in Yr-1. In the much drier Yr-2 far fewer flower-heads were available throughout the entire extent of the population. This obliged us to undertake the pollination observations in the upper reaches of the lower area where flowering individuals were somewhat more abundant than on the better-drained upper slopes and vice-versa use the upper slopes and rest of the population throughout its entire extent for extractive and other requirements. In both years we observed dry stems of *M. subulata*, with greater drying in Yr-2.

### 4.3. Characterization of the Central Chilean Mega Drought

No weather station data are available for the specific study area. However, the regional climate and annual anomalies can be estimated using data from nearby stations with long-term records. The closest station to our study site with a long-term record is Quinta Normal in the city of Santiago. We obtained temperature and precipitation for Quinta Normal from the CR2 Climate Explorer, available at http://explorador.cr2.cl/. To further characterize the drought, we obtained the daily elevation of the snow-line using the MODIS/Terra and Aqua Snow Cover Daily L3 Global 500m Grid (products MOD10A1 and MYD10A1) (https://worldview.earthdata.nasa.gov/). Pixels with more than 80% of snow were considered snow-covered and their elevation was derived from the Shuttle Radar Topography dataset. The snow line for each day corresponds to the lowest 1% elevation of the snow-covered pixels [13].

### 4.4. Floral Traits

To evaluate flower-head appearance to hummingbirds, flower colour was represented in the avian tetrahedral colour space [100]. For this, a spectrometer (Ocean Optics USD2000, Dunedin, FL, USA) was used to measure the spectral reflectance of rays obtained from multiple flower-heads (N = 30), leaves (N = 30) and stones (N = 15) against which the flower-head must be detected by the birds. The samples were transported to the laboratory and illuminated with a deuterium-halogen lamp (Avantes AvaLight-DH-S) through an optical fiber, while a second fiber connected to the spectrometer collected the light reflected by the sample. A white reference standard (Avantes WS-2) was used to calibrate the spectrometer. The spectral reflectance of the samples was then plotted in a tetrahedral colour space modelled using the spectral sensitivity of birds’ cone type photoreceptors, as described by [101]. The avian tetrahedral space was modeled considering the standard avian colour vision system with VS, S, M and L type cones which correspond to cones maximally sensitive to violet, blue, green and light red, respectively. The analyses were performed using the PAVO package [102].

In Yr-1, flower-head longevity was monitored on 25 pollinator-excluded heads in the field (1 per plant) from the day the tips of the rays florets began breaking out until they finally closed or were about to close. The marked heads were enclosed in transparent pollination bags to exclude pollinator visitation. All heads were checked daily around midday with the following stages recorded: (1) ray florets appearing and unfolding up to the stage where all disk florets remain closed; (2) appearance of the first disk florets extruding pollen through to the last florets with bifurcate fresh stigmas; (3) stigmas of all disk florets drying out; (4) ray florets beginning to close or completely closed. To determine the life-span of individual florets, we marked unopened samples in the flower-heads with a permanent marker and monitored them daily according to the following stages: (1) pollen extruded from corolla; (2) stigma exserted but not yet bifurcate; (3) stigma bifurcate; (4) stigma drying out. It was not possible to measure flower-head longevity in a large sample of flower-heads in Yr-2 given the lack of available flower-heads. That year we mounted four time-lapse cameras (Brinno) (1 image per hour) inside wire bird-cages covered with muslin to exclude pollinators that focused on a total of six flower-heads. Unfortunately, most of the flower-heads mysteriously disappeared over the coming weeks possibly due to florivory by a nocturnal lizard that dwells on the slopes and managed to slide under the bird cages.

### 4.5. Pollinator Observations

Data on visitation frequency (flower-heads visits/plot/unit time) were compiled by four observers working simultaneously on five and four separate days over the periods 18–23 February (2019) (Yr-1) and 18–21 February (2020) (Yr-2), respectively, starting at 7.00 h and ending at 19.00 h (standard time) each day. In both years, four 15 m × 15 m quadrats were established (total of 900 m^2^). On each plot, observations on flower visitors were made during three 15 min periods per hour, intercalated with two 5 min breaks to minimize fatigue (total of 45 min observation time per hour on each plot). To counteract the observer effect, plots were continuously rotated. Over each 15 min observation period, for hummingbirds, butterflies and bees, we recorded: (1) each time a flower-head was visited and visitor identity; (2) where visually feasible, the number of florets probed per flower-head visited. Additional data on florets probed were obtained on 17 February in the first year while assessing adequate plot size for the pollinator observations. During the first day of Yr-1, it became evident that some of the hummingbird visits took place behind rocks as the birds descended rapidly down close to ground level and thus were not always completely visible for an observer´s post. Therefore we separated visits that could be easily seen from those that were inferred from the peculiar behaviour of the birds in order to determine if the two measures of visitation were correlated. On the flatter part of the population where the pollinator observations were made in the second year (which had few and smaller rocks) observing visitation was much easier. We could have missed some of the visits behind rocks in Yr-1 because we always recorded these as a single flower-head visit. In addition to the observations mentioned above, the observers mostly recorded flight bouts into the plots by anthophyllous animals that did not or hardly visited the flower-heads. This last metric was not maintained for bees which were rarely seen on the plots. To ascertain that the orange-yellow pollen on the heads of the birds belonged to *M. subulata*, we scraped pollen off one mist-netted hummingbird in Yr-1 for microscopic examination.

### 4.6. Floral Resource Availability

We obtained two measures of floral resource availability. First, on each pollinator observation day, the number of open flower-heads was counted at a plot-level. These data were also used to calculate the number of flower-head visits per hour (visitation rates).

In both years we sampled nectar standing crop at two hourly intervals over 5 (Yr-1) (17, 18, 20, 22, 23 February) and 4 (Yr-2) (18–21 February) days according to the following procedure. Every two hours starting at 7 a.m. and ending at 5 p.m. standard time, one head per plant for five separate plants was harvested in the field and transported rapidly in a cooler to a field laboratory. Nectar was extracted from a targeted five individual florets per flower-head covering both the male and female stages with 0.5 or 1.0 lambda microcapillaries. To avoid evaporation, all nectar was extracted within the same two hour bracket. Because extraction from the very narrow florets was slow, it was impossible to obtain nectar standing crop at hourly intervals. The latter would have signified spending much more time travelling back and forth between the site and the field laboratory. In Yr-2 when there were fewer available flower-heads, nectar standing crop was obtained for 3 flower-heads per every two hours increasing the floret number per head. Sampling over a complete day was considered important—nectar production in Mediterranean-type climate plants has been shown to be negatively affected by high temperatures which will occur in the midday hours and which could be expected to affect the daily course of flower visitation in exceptionally warm years [42,103]. To assess potential accumulated nectar we undertook the same procedure on a different set of flower-heads over a single day considering 3 plants per every two hours in Yr-1 and 3 plants per every four hours in Yr-2. These heads were previously pollinator-excluded. Although we tried, it was impossible to obtain the small amounts of what was fairly concentrated nectar out of the microcapillaries onto a hand-held refractometer to measure its sugar content in the field. After the pollination survey was completed we transported a new set of flower-heads harvested again at different times of the day on ice packs back to the laboratory in Santiago to try a different procedure for determining the sugar content of the nectar. Nectar was extracted from individual florets with a 0–20 µL micropipette and deposited in Eppendorf tubes that contained 30 µL of distilled water. The solution of water and nectar was then collected in a 0–200 µL micropipette to obtain the total volume and the volume of the nectar by subtraction. The sugar concentration of the solution (weight/weight) was measured with a standard hand-held refractometer (0–32 Brix, resolution 0.1 Brix). We then calculated the sugar concentration in the nectar with the standard formula C_1_V_1_ = C_2_V_2_ where C_1_ and V_1_ are the concentration of sugar in the solution of nectar and water, and its volume, respectively, and C_2_, V_2_ are the sugar concentration and volume of the nectar. In order to discard potential errors produced by very small nectar volumes, we discarded samples where less than 5 µL of nectar had been added to the 30 µL of water. In Yr-2, due to the acute lack of flower-heads, nectar concentration could only be established for a few flowers-heads collected at different time of the day. That year the smaller number of heads was compensated for by sampling more florets per head using an original volume of 60 µL distilled water.

### 4.7. Seed Set

To determine the extent to which *M. subulata* requires external pollination for seed set, the same 25 pollinator-excluded flower-heads (1 per plant) followed for flower-head longevity were checked for seed formation several weeks after the heads had closed. To obtain a measure of open-pollination seed set in the two years, achene production per flower-head was determined for the pollinator-observation areas each year. In Yr-1, towards the end of the pollinator sampling period, we haphazardly marked a large sample of flower-heads in the four plots that still remained open or had recently closed. Several days later when the flower-heads were closed or were about to completely close, they were individually bagged to capture seeds. In Yr-2 when flower-heads were scarce, we marked all heads that were open in the pollination plots and added additional heads after the pollinator sampling was complete in order to obtain a reasonable sample size. The bagged flower-heads were retrieved on different dates in late March and early April for the purposes of counting seed. All good seed has been saved for return to the study site.

### 4.8. Onsite Abiotic Variables

Hourly temperature and relative humidity at 1.5 m.a.g.l were obtained from an automatic battery-powered recording device (HOBO U23 Pro v2; Onset Computer Corp., Cape Cod, MA, USA) fitted with a custom-made sun shield, which had been set up some 100 m from the study site in September, 2018. The downloaded data allowed characterization of temperature conditions and relative humidity on the site as of snow melt and for the specific days and hours of the pollinator observations each year. Instantaneous wind speed was recorded at 1.5 m.a.g.l. in two of the pollinator observation plots in Yr-1. In Yr-2, given that the elevation of the plots showed little variation, a single anemometer was set up. At the beginning and end of each 15 min observation period, wind speed (kph) was recorded to give six and three pairs of values per hour in 2019 and 2020, respectively. Cloud cover was recorded according to three categories (cloudless, partially cloudy, heavy clouds) during each 15 min observation.

### 4.9. Data Analysis

**Figure A1:**
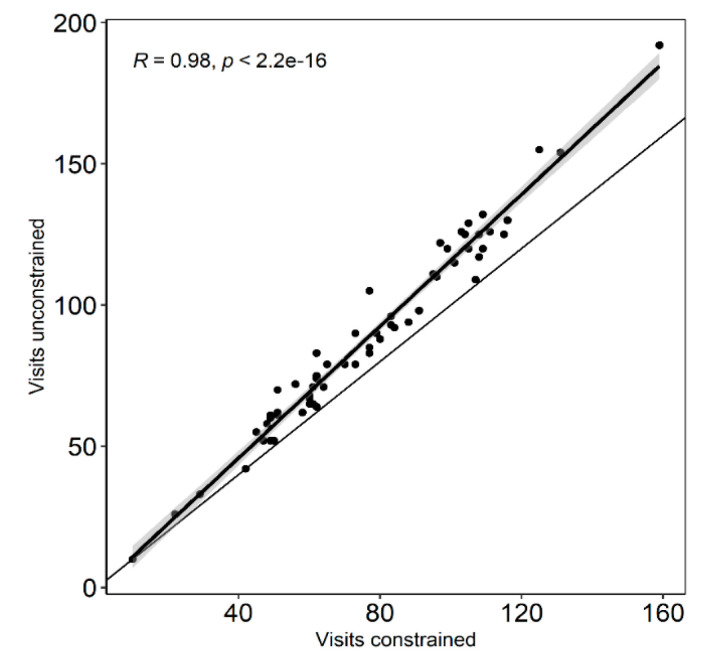
Relationship between total number of visits to flower-heads by hummingbirds including visits behind rocks (visits unconstrained) and the number based only on flower-head visits that could be easily seen (visits constrained). The data points are the sum of the visits recorded on the four plots per hour over each day (5) of the pollinator sampling (N = 60). The fine black line shows the 1:1 expectation.

**Figure A2:**
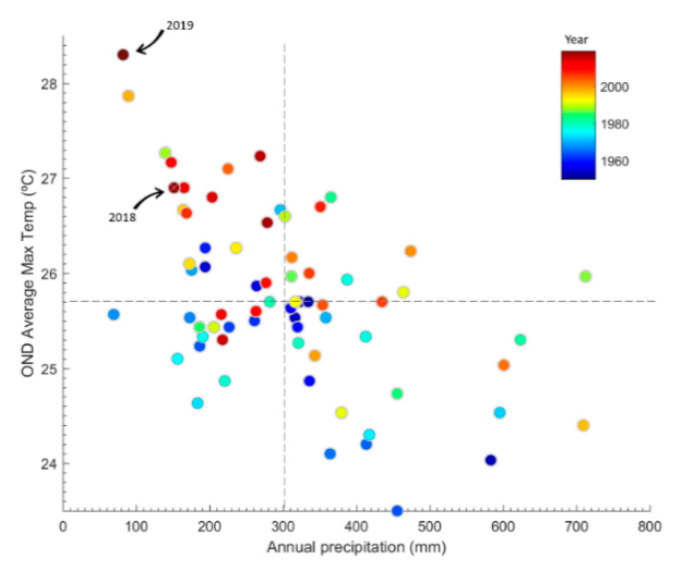
Relationship between mean annual precipitation (mostly concentrated in May.–Jun.–Jul.–Aug.) and mean maximum temperature for Oct.–Nov.–Dec. as of 1950 in central Chile based on weather data for Santiago (Quinta Normal). The figure shows that warmer than average conditions in late spring/early summer tend to be more frequent during drier than average winters (*r* ~ −0.5, *p* < 0.01). The dashed lines indicate the long-term median values. The filled circles are coloured according to year, from 1950 (dark blue) to 2019 (dark red). The circles indicating 2018 and 2019 are the spring-early summer months prior to the February Yr-1 and Yr-2 pollinator sampling dates. Data from Dirección Meteorológica de Chile (DMC) obtained via the CR2 climate explorer: http://explorador.cr2.cl/.

The two measures of hummingbird visitation taking into account visits behind rocks versus without inclusion of the latter (as explained in Section 4.5) were highly correlated (see Figure A1), thus for all analyses we used the data for all visits recorded. Flower-head visitation rates were calculated as the total number of visits per hour divided by the number of open flower-heads. As the number of visits, visitation rates and nectar standing crop volume per floret were not normally distributed we performed aligned rank transforms for nonparametric factorial analyses with the *ART* package of R v.4.0.2 [104] to test for main effects and interactions of year and period of day using ANOVA procedures. The beginning times for the three periods of the day considered in the visitation frequency and visitation rates analyses were: 7 a.m., 8 a.m., 9 a.m. and 10 a.m. (morning), 11 a.m., 12 a.m., 1 p.m. and 2 p.m. (midday) and 3 p.m., 4 p.m., 5 p.m. and 6 p.m. (afternoon). For nectar standing crop where nectar could only be harvested at two hourly intervals, the corresponding blocks started at 7 a.m. and 9 a.m., 11 a.m. and 1 p.m. and 3 p.m. and 5 p.m. Dunn’s test for multiple comparisons using rank sums was used to test for differences in the two visitation measures over the day and among years. For these analyses we used the hourly values (data for 4 plots combined) across all observation days (Yr-1, N = 80; Yr-2, N = 64). For nectar standing crop the unit of comparison was the individual floret. Sample sizes varied between 186 and 247 florets extracted per the three periods of the day. Differences for flower-head number per plot, flower-head diameter, number of ray and disk florets per flower-head, number of florets probed per flower-head visited, accumulated nectar per floret and seed set among years were tested using the Mann–Whitney U-test for non-parametric data in R.

To evaluate the effect of direct and indirect drivers on pollinator visitation, we conducted linear and generalised linear mixed models using different combinations of the following variables: cloud cover, temperature, wind speed, relative humidity, nectar standing crop and flower-head density. We chose the model structure providing the lowest Akaike information criterion using the *stepAIC* function in the “MASS” package of R in order to lower complexity but maintaining a good fit [105]. We checked for collinearity between variables using the *vif* function in the “car” package of R [106]. Values of VIF < 5 indicate non-concerning collinearity. The raw data were the hourly values per sampling plot (as replicates) for each day and year. Visitation data and flower-head number were available for each individual sampling plot. Hourly wind speed was obtained by averaging data collected for each 15 min observation period per hour. Cloud cover for each hour was represented on a scale of three 3: 1 = completely clear; 2 = partially cloudy; 3 = heavy cloud. Hourly temperature was the mean of three values per hour corresponding to the beginning of each 15 min observation period. The first value was the recording device value for the respective hour. The second and third were obtained by interpolation between two consecutive hours. Nectar standing crop was available at two hourly intervals. To estimate nectar standing crop for the intervening hours, we interpolated between the pairs of consecutive hours. As mentioned previously (see Section 4.6), logistic considerations (time required to collect the flower-heads, travel to and from the field laboratory, and extract nectar from the many florets) only allowed us to determine nectar standing crop at two hourly intervals. For the last hour of the day we used the same value as that of the previous hour. For cloud cover, temperature, wind speed, relative humidity and nectar standing crop we used the same hourly values for each of the four sampling plots. These last variables are not expected to vary measurably within an area of 900 m^2^. The complete data set used to obtain the models can be found in Table S1.

## 5. Conclusions

Looking for general rules regarding the long-term impacts of drought and global warming on plant–pollinator interactions is daunting, given that the main groups of animal pollinators and the plants they pollinate are likely to be affected by warming and drier conditions in different ways. To our knowledge, our study is the first attempt to sort out the effects of a number of potential direct and indirect drivers of pollinator visitation and hence, seed set in a natural population setting. The overwhelming effect of floral density on visitation was not unexpected and probably has more than one cause. We came up with some surprises: hummingbird visitation was positively affected by higher ambient temperatures but nectar standing crop was not a significant effect.

In retrospect, our work would have benefited from more pollinator observation days spread over the entire flowering season. This is easier said than done. Sorting out the relative contributions of direct and indirect effects of any expression of climate change on pollination systems in natural populations is not a simple task given the enormous amount of data that must be obtained on each sampling day under less than optimal working conditions. Experimental watering using potted plants or small vegetation quadrats are good alternatives, but even then, controlling for factors like pollinator mortality and relocation of pollinators to other plant species will always be difficult. For both approaches, long-term studies constitute the only realistic way to separate out the effect of human-driven temperature increases and a temperature increase that might arise as a physical side effect of a precipitation deficit in a given year.

It could be argued that plant–pollinator systems in Mediterranean-type climate ecosystems are adapted to drought in which case effects of the nature documented here will be transient and thus insignificant in the long term. However, with longer and more severe droughts expected with increasing temperatures and declining precipitation over this century in central Chile, the possibility of tipping points being reached in the future cannot be discarded. Clearly, the interplay between precipitation and temperature is critical for understanding the effects of climate change on plant–pollinator interactions and plant fitness in Mediterranean-type climate ecosystems.

Finally, the colour, size, and prolonged longevity of the flower-heads of the hummingbird pollinated species studied, together with the reward they provide, were seen to engender a high level of structural correspondence between the two mutualists leading to a specialised interaction. It is not at all surprising, therefore, that flower-head density and relative humidity (considered a proxy for nectar sugar concentration) turned out to be the main drivers of pollinator visitation.

## Figures and Tables

**Table 1:** Total visits by hummingbirds and butterflies recorded on *M. subulata* flower-heads over five days in Yr-1 and 4 days in Yr-2 on four plots summing 900 m^2^ each year in the central Chilean Andes, 2450–2500 m.a.s.l. Additionally given are the total flight bouts of hummingbirds and butterflies into the sampling areas irrespective of whether visits to *M. subulata* were made. No other plant species was visited by hummingbirds in the pollinator observation plots in either year. Butterflies visited *Senecio pentaphyllus*. Data are for the same week of February each year. Three visits (not shown) were made by a single bumblebee species in February 2019.

Pollinators	Year	Total Flight Bouts Recorded on Plots	Total Flight Bouts with Visits	Total Flower-heads Visited
			N (%)	
Hummingbirds	Yr-1	2131	1119 (52.5%)	5345
	Yr-2	55	44 (80.0%)	117
Butterflies	Yr-1	1955	12 (0.6%)	13
	Yr-2	2679	7 (0.3%)	10

**Table 2:** Results of the nonparametric factorial analysis (Aligned rank transformed) for the number of visits to *M. subulata* flower-heads and flower-head visitation rates considering the last two years of the Chilean mega drought. Significant effects are shown in bold.

**Number of Flower-Head Visits**				
	Sum Sq	Df	F value	Pr (>F)
**Year**	4,316,679.0	1	832.66	**2.88 × 10^−102^**
**Period of day**	663,385.2	2	24.39	**9.39 × 10^−11^**
**Year × Period of day**	436,201.3	2	15.34	**3.68 × 10^−07^**
**Flower-Head Visitation Rate**				
	Sum Sq	Df	F value	Pr (>F)
**Year**	1,667,083.4	1	145.57	**4.90 × 10^−29^**
**Period of day**	365,901.8	2	13.20	**2.74 × 10^−06^**
**Year × Period of day**	103,221.6	2	3.57	**2.89 × 10^−2^**

**Table 3:** Results of nonparametric factorial analysis (aligned ranks transforms) for nectar standing crop in *M. subulata* over the last two years of the Chilean mega drought. Significant effects are shown in bold.

	Sum Sq	Df	F Value	Pr (>F)
Year	11,711,806.5	1	89.93	**1.13 × 10** **^−^** **^20^**
Time of day	860,276.3	2	3.36	**3.52 × 10** **^−^** **^2^**
Year × Time of day	1,414,651.0	2	5.50	**4.00 × 10** **^−^** **^3^**

**Table 4:** Results of the linear model (LM) considering direct and indirect drivers of visitation frequency. The best model and its statistics can be found on the first line of the table: VF = visitation frequency, NSC = nectar standing crop, T = temperature, RH = relative humidity, FHD = flower-head density. The statistics for the individual variables of the best model are given in the rows that follow. Significant values are shown in bold.

Model: VF ~ Intercept + NSC + T + RH + FHD		r^2^ = 0.4561		*p* < 0.0001
Variable	Estimate	SE	T	*p*
Intercept	−21.913	6.853	−3.198	**0.0015**
Nectar standing crop	2.883	4.535	0.636	0.5253
Temperature	0.616	0.239	2.572	**0.0105**
Relative humidity	0.326	0.084	3.884	**0.0001**
Flower-head density	28.945	4.053	7.142	**<0.0001**

## Supplementary Materials

The following are available online at https://www.mdpi.com/2223-7747/9/11/1553/s1, Table S1: Data used to model the effect of several variables on visitation frequency (number of visits on plots of equal size). Video S1: *Oreotrochilus leucopleurus* visiting the subalpine form of *Mutisia subulata*, 2450 m.a.s.l. in the Farellones area, central Chile. Captured originally on 22 February, 2019. Credit: Ana Copier. If you wish to reproduce this video for educational purposes please give it due credit and cite this publication; Video S2: *Oreotrochilus leucopleurus* visiting the subalpine form of *M. subulata*, 2450 m.a.s.l. in the Farellones area, central Chile. Captured originally on 3 March 2019. Credit: Mary T. K. Arroyo. If you wish to reproduce this video for educational purposes please give it due credit and cite this publication.
